# Proteomic Profiling of Immune Cell Infiltration Reveals Prognostic Immune Subtypes in Lung Adenocarcinoma

**DOI:** 10.3390/proteomes14040052

**Published:** 2026-10-02

**Authors:** Ruoxian Zhang, Hongsheng Guo, Xianju Li, Hongjie Zhao, Jun Qin

**Affiliations:** 1 Institutes of Biomedical Sciences, Fudan University, Shanghai 200032, China; 22111510042@m.fudan.edu.cn; 2 Department of Medical Oncology, Central Hospital, Tianjin University/Third Central Hospital, Jintang Road 83, Hedong District, Tianjin 300170, China; 3 Beijing Proteome Research Center, Beijing 102206, China; 4 Department of Thoracic Surgery, Baodi Hospital, Tianjin Medical University, Tianjin 301000, China

**Keywords:** lung adenocarcinoma, proteomics, immune infiltration, immune subtype, disease-free survival, CPTAC, tumor microenvironment

## Abstract

Background: Lung adenocarcinoma (LUAD) shows marked heterogeneity in its immune microenvironment, which may contribute to differences in disease-free survival (DFS) and clinical outcome. Although current LUAD classifications are largely based on genomic or transcriptomic features, immune subtypes defined directly from bulk proteomic data remain poorly characterized. Here, we used an immune-cell proteomic reference matrix to investigate proteome-defined immune states in LUAD and to identify prognostically relevant immune subtypes. Methods: A quantitative human immune-cell proteomic reference matrix was integrated with LUAD tumor proteomic profiles. The reference matrix covered seven major immune lineages and 26 immune-cell states. The discovery cohort included 101 LUAD tumor proteomes with matched clinical information. After gene symbol matching and normalization, proteome-defined immune-cell state signals were estimated for each sample using non-negative constrained regression. These signals were then used for unsupervised subtype identification and DFS analysis. An independent Clinical Proteomic Tumor Analysis Consortium (CPTAC)-LUAD cohort was used for validation, with subtype analysis performed at the immune-cell marker protein module level. Results: The immune-cell reference matrix contained 8400 quantified proteins, and 2381 proteins were retained for modeling. Based on 26 proteome-defined immune-cell state signals, LUAD samples separated into two immune proteomic subtypes. In the discovery cohort, Subtype 2 had significantly worse DFS than Subtype 1 (log-rank *p* = 0.0047; HR = 2.09, 95% CI 1.24–3.54). In the Clinical Proteomic Tumor Analysis Consortium (CPTAC)-LUAD validation cohort, marker protein module-based analysis again identified two subtypes with distinct DFS outcomes. The high-risk Subtype 2 showed higher Granulocyte and Myeloid/DC modules, whereas the low-risk Subtype 1 showed higher B cell, T cell, and NK-cell modules. Subtype 2 was associated with shorter DFS in the validation cohort (log-rank *p* = 0.0061; HR = 3.45, 95% CI: 1.35–8.82), and this association remained significant after adjustment for age, sex, and stage. Conclusions: Proteomic immune profiling identified two prognostically distinct immune subtypes in LUAD. The poor-DFS subtype was characterized by increased myeloid/granulocyte-related protein modules, whereas the favorable-DFS subtype showed stronger B cell-, T cell-, and NK cell-related signals. These findings support the use of bulk tumor proteomics to characterize clinically relevant immune heterogeneity in LUAD.

## 1. Introduction

Lung adenocarcinoma (LUAD) is one of the most common histological subtypes of non-small-cell lung cancer (NSCLC) and a major cause of cancer-related deaths. With advances in genomics and targeted therapies, significant progress has been made in molecular subtyping, prognostic modeling, and treatment strategies for LUAD [1,2]; however, patients with similar driver genes or comparable clinical stages may still exhibit markedly different disease-free survival outcomes and clinical prognoses. This suggests that the clinical heterogeneity of LUAD is determined not only by intrinsic molecular alterations in tumor cells but is also profoundly influenced by the tumor microenvironment—particularly the state of the immune microenvironment and its interaction with stromal components [3].

The tumor immune microenvironment plays a critical role in tumor initiation, progression, treatment response, and prognosis. Previous studies have shown that the composition and functional state of immune cells within tumor tissue—known as the “immune contexture”—are closely associated with clinical outcomes in various solid tumors [4,5]. In early-stage EGFR-mutant LUAD, tertiary lymphoid structures and immune microenvironment signatures have also been associated with clinical outcome [6]. In the tumor immune cycle, processes such as antigen release, antigen presentation, T-cell activation, immune cell recruitment, and tumor cell killing collectively determine the efficacy of the antitumor immune response [7]. Pan-cancer studies have further demonstrated that significant differences in the immune landscape exist across different tumor types and among patients, and that patterns of immune cell infiltration can reflect tumor immune activity, immunosuppressive states, and potential treatment sensitivity [8,9]. In lung adenocarcinoma (LUAD), immune signals associated with T cells, B cells, and natural killer (NK) cells are typically linked to stronger antitumor immune responses, whereas myeloid cells, granulocytes, and immunosuppressive inflammatory states may contribute to immune evasion, tumor progression, and poor disease-free survival (DFS) outcomes [1,7]. Therefore, systematically analyzing the immune cell infiltration patterns in LUAD and identifying immunological subtypes with prognostic significance are crucial for risk stratification and understanding the immunobiological mechanisms of lung adenocarcinoma.

Currently, the analysis of immune cell infiltration in whole tumor samples primarily relies on transcriptomic data. Methods such as CIBERSORT, CIBERSORTx, MCP-counter, xCell, quanTIseq, EPIC, and ESTIMATE have been widely used to infer the composition of immune or stromal cells from whole-tumor RNA expression matrices [10,11,12,13,14,15,16,17]. These methods have greatly advanced research on the tumor immune landscape, enabling researchers to assess immune cell infiltration and tumor purity in large-scale public transcriptomic cohorts. However, mRNA abundance does not always correlate with protein abundance. Proteomic measurements directly quantify protein-level signals, but the extent to which proteoforms are resolved depends on the experimental platform, peptide-level evidence, database-search strategy, and available annotation. In the present study, the immune-cell reference and LUAD tumor matrices were analyzed primarily at the canonical protein/gene-symbol level. Although the source proteogenomic studies may contain peptide- or modification-level information, proteoforms were not systematically resolved or modeled in the current analysis. Therefore, the inferred immune-cell-state signals should be interpreted as canonical protein-level signatures rather than proteoform-resolved measurements. Distinct proteoforms, including post-translationally modified or alternatively processed forms, may have different cellular distributions or functional activities and could potentially influence immune-state estimation. Future analyses integrating peptide-level evidence, phosphoproteomics, and proteoform-resolved measurements may refine the reference matrix and improve the biological resolution of proteomic immune subtyping [5]. Proteins are the direct effector molecules involved in antigen presentation, cytotoxicity, inflammatory responses, chemokine signaling, and immune regulation; therefore, proteomic data may provide immune information that more closely reflects functional states [18,19,20]. With the continuous accumulation of mass spectrometry proteomics and proteogenomics cohorts, it has become possible to analyze immune cell infiltration and immune status directly from the overall tumor proteome.

In recent years, human immune cell proteomic reference datasets have provided a crucial foundation for analyzing immune states at the proteomic level. Rieckmann et al. systematically mapped the protein abundance networks of human immune cells using quantitative proteomics, demonstrating that different immune cell lineages and cellular states exhibit distinguishable protein abundance profiles [21]. Such immune cell proteomic reference matrices enable the mapping of bulk tumor proteomes to the immune cell state space, thereby allowing for the estimation of the relative intensity of protein signals associated with different immune cell states. Meanwhile, public LUAD proteomics cohorts provide a data foundation for such analyses. The lung adenocarcinoma proteogenomics cohort reported by Xu et al. includes 101 LUAD tumor proteomic samples and matched clinical information [18]; the CPTAC-LUAD cohort further provides an independent processed tumor proteome matrix and clinical follow-up information, which can be used for external validation [22].

Based on the above background, this study aims to analyze the characteristics of immune cell infiltration in LUAD at the proteomic level and to identify immune subtypes with prognostic significance. We integrated a human immune cell protein reference matrix with LUAD bulk tumor proteomic data and, through gene symbol matching, matrix normalization, and non-negative constrained regression, calculated protein-defined immune cell status signals for each tumor sample. Subsequently, we performed unsupervised clustering of LUAD samples based on a protein-based immune profile comprising 26 immune-cell statuses and assessed its association with disease-free survival (DFS). We performed external validation in an independent CPTAC-LUAD cohort using a strategy based on immune cell marker protein modules. This study demonstrates that identifiable patterns of immune cell infiltration exist in the LUAD proteome and that these patterns can define two immune subtypes with distinct prognostic and immunoproteomic characteristics, providing a new basis for proteomic-level immunotyping and DFS risk stratification in lung adenocarcinoma.

## 2. Materials and Methods

### 2.1. Data Sources

This study utilized publicly available proteomics data and clinical annotation information. The present analysis used gene-symbol-level protein abundance matrices derived from the source proteomic studies. Proteoforms and site-specific post-translational modifications were not systematically resolved or modeled. The reference proteomic data for immune cells were derived from a quantitative proteomics study of human immune cells reported by Rieckmann et al. [21], with the corresponding ProteomeXchange/PRIDE (EMBL-EBI, Hinxton, UK)accession number PXD004352. The LUAD discovery cohort was derived from the lung adenocarcinoma proteogenomics study reported by Xu et al. [23], with the corresponding ProteomeXchange accession number PXD020252. The external validation cohort utilized the CPTAC-LUAD (NCI Proteomic Data Commons, study accession PDC000153; https://proteomic.datacommons.cancer.gov/pdc/study/PDC000153, accessed on 22 September 2026) processed proteomics data [19].

### 2.2. Processing of Immune-Cell Proteomic Data

Raw mass spectrometry files from immune cells were processed using the Firmiana platform (version 1.0; Beijing Proteome Research Center, Beijing, China) [16]. Database searches were performed using the MASCOT search engine (version 2.3; Matrix Science Ltd., London, UK) [24], with the NCBI Human RefSeq Protein Database (2013 release, 32,015 entries; National Center for Biotechnology Information, Bethesda, MD, USA) as the reference database. The search parameters were as follows: parent ion mass tolerance of 20 ppm; daughter ion mass tolerance of 0.5 Da; trypsin digestion with a maximum of 2 cleavage sites allowed; cysteine formylation set as a fixed modification; and N-terminal acetylation and methionine oxidation set as variable modifications.

The false discovery rate (FDR) for peptide identification was set to 1%. During protein assembly, proteins meeting any of the following criteria were retained: at least 1 unique peptide; at least 2 high-quality peptides; at least 3 high-quality peptides; or 1 unique peptide with an ion score > 40. Protein quantification was performed using the intensity-based absolute quantification (iBAQ) method [20]. The iBAQ value for each protein was divided by the sum of the iBAQ values of all identified proteins in that sample to obtain the “fraction of total”; this value was then multiplied by 10^5^ to obtain the iFOT value. Missing values in the raw quantification matrix were treated as 0.

### 2.3. Construction of the Immune-Cell Protein Signature Matrix

The immune-cell reference dataset comprised quantitative proteomic profiles of 104 samples representing 26 immune-cell states, with four replicate samples per state. The original sample-level matrix contained 8400 proteins represented by gene symbols. Proteins with non-zero abundance values in all four replicates of at least one immune-cell state were retained as candidates, yielding 6122 candidate proteins. These candidates were subsequently reviewed manually based on their functional annotations, and 2381 proteins were retained based on their immune relevance.

For each retained protein, its reference abundance in each immune-cell state was calculated as the arithmetic mean across the four corresponding replicate samples, yielding a 2381 × 26 reference matrix. Rows represented proteins, and columns represented immune-cell states. All 2381 gene symbols were verified to be present in the original sample-level matrix, and all reference-matrix values were confirmed to match the corresponding replicate means. The reference matrix was subsequently matched to the tumor proteomic matrix and standardized as described in Section 2.4.

### 2.4. Proteomic Estimation of Immune Infiltration

The bulk LUAD tumor protein abundance matrix is regarded as a mixture matrix composed of protein abundance signals corresponding to different immune cell states. For each tumor sample, let its protein abundance vector be M, the immune cell protein signature matrix be S, and the immune cell state weights be F. Then, the deconvolution model is expressed as follows:

*M = S × F*

The bulk tumor mixture matrix was first quantile-normalized across samples using the preprocessCore::normalize.quantiles function and was then intersected with the immune-cell signature matrix at the gene-symbol level. Gene symbols were trimmed, converted to a common case, and duplicate gene symbols were collapsed by taking the mean abundance across samples before intersection. Within the shared protein feature space, the immune-cell signature matrix was globally standardized using the overall mean and standard deviation across all proteins and immune-cell states. Each tumor-sample abundance vector was independently z-score standardized across the shared proteins. Non-negative regression coefficients were estimated using quadratic programming with coefficients constrained to be greater than or equal to zero. The fitted coefficients were subsequently normalized within each sample so that the sum of the 26 immune-cell-state signals equaled one.

The proteomic immune-cell state signal represents the relative protein signal in the bulk tumor proteome that matches the corresponding immune-cell state signature pattern; it is not interpreted as an absolute cell proportion. The quality of the model fit is evaluated using Pearson correlation and root mean square error. These fit quality metrics are used to assess model quality and do not serve as criteria for sample exclusion.

### 2.5. Identification of Immune Subtypes

Unsupervised clustering was performed using a custom Brunet-like non-negative matrix factorization algorithm implemented in R 4.4.2 [25,26]. A rank-2 model was prespecified to identify two broad immune subtypes and to maintain the same subtype dimensionality in the discovery and validation cohorts. The input matrix contained only the 26 non-negative proteomic immune-cell-state signals from the 101 LUAD samples. The analysis used rank 2, 100 independent runs, a maximum of 500 multiplicative-update iterations per run, and a fixed initial random seed of 123. The random seed was incremented by one for each run. The solution with the lowest sum of squared reconstruction error was selected as the final model. Each sample was assigned to the subtype corresponding to the NMF component with the larger sample-specific coefficient. “Subtype 1” and “Subtype 2” are analytical labels and do not represent pre-specified biological categories.

### 2.6. External Validation Cohort

The external validation cohort utilized CPTAC-LUAD proteomics data [19]. The protein abundance matrix was converted to the gene symbol level using the CPTAC-LUAD gene annotation/ID mapping file, and then intersected with the immune cell protein signature matrix. Duplicate gene symbols were merged based on the mean abundance across samples. Samples with available DFS information were included in the survival analysis.

Recurrence-free survival, recurrence status, and follow-up information from the raw CPTAC-LUAD clinical annotations were uniformly consolidated into the DFS endpoint, as these fields all reflect the time from initial treatment to a recurrence event or censoring. For samples with an event, the event time was used as the DFS time; for samples without an event, the time of last follow-up, available recurrence-free survival time, or available total follow-up time was used as the censoring time. The term “DFS” is used consistently throughout the text.

### 2.7. Marker Protein Module Analysis in the Validation Cohort

Based on cell-state-specific proteins in the immune cell signature matrix, immune-related proteins were grouped into five categories of marker protein modules, as follows: B cell, T cell, NK, Myeloid/DC, and Granulocyte. Each module prioritized the retention of marker proteins detectable in the CPTAC-LUAD processed protein matrix. For each module, up to the top 30 ranked marker proteins were considered, and proteins not detected in the CPTAC-LUAD processed matrix were excluded. For each retained protein, abundance was z-score standardized across samples, and the module score was calculated as the mean of the retained protein z-scores.

Before NMF, each module was z-score standardized across samples. The resulting five-module score matrix was then shifted by subtracting its global minimum and adding 1 × 10^−8^ to ensure strictly positive input values. The NMF parameters are consistent with those used in the discovery cohort. The resulting subtypes are used for DFS analysis and comparisons of marker module differences. To evaluate external validation strategies, marker module NMF, marker module hierarchical clustering, overall marker score median split, and single marker module median split were all included in the comparison. In the main text, marker module NMF is adopted as the primary external validation strategy.

The five-module representation was used as a practical compromise between immune-lineage resolution, protein detectability, and biological interpretability. Several markers associated with finer immune-cell states were not consistently detected in the CPTAC-LUAD proteomic matrix, limiting their use as separate validation modules. This strategy therefore provided external validation at the broad immune-lineage level rather than a direct replication of all 26 immune-cell states used in the discovery analysis.

### 2.8. Survival Analysis

The Kaplan–Meier method was used to estimate DFS curves for the different immune subtypes. Differences between subtypes were assessed using the two-sided log-rank test. Cox proportional hazards regression was used to estimate hazard ratios (HRs) and 95% confidence intervals (CIs) [27]. In the discovery cohort, univariable Cox models were first constructed, followed by multivariable models adjusted for age, sex, and TNM stage. Additional models further included smoking status, EGFR mutation, TP53 mutation, and KRAS mutation. In the validation cohort, multivariable Cox models were adjusted for age, sex, and pathological stage. All Cox models used Subtype 1 as the reference group.

For all survival analyses, a DFS status of 1 indicated a documented recurrence or other DFS event, whereas a DFS status of 0 indicated censoring. DFS follow-up times were analyzed in months. Samples with missing or non-finite DFS times, missing event status, or DFS times of zero or less were excluded from the corresponding survival analysis. Cox models were fitted using complete cases for the variables included in each model; samples with missing covariate values were omitted from the corresponding model.

### 2.9. Statistical Analysis

Comparisons of continuous variables between the two groups were performed using the two-sided Mann–Whitney U test. Macrophage-associated proteins were evaluated individually when detected in the LUAD proteomic matrix, including CD80, CD86, CD163, and MRC1, the official gene symbol for CD206. Intergroup comparisons of multiple immune cell states or marker protein modules were adjusted for the false discovery rate (FDR) using the Benjamini-Hochberg method [28]. Unless otherwise specified, an FDR < 0.05 was considered statistically significant after correction for multiple comparisons. Similarity between samples was assessed using Pearson correlation. All statistical tests were two-sided; *p* < 0.05 was considered statistically significant.

All analyses were performed using R (version 4.4.2; R Foundation for Statistical Computing, Vienna, Austria). The primary R packages used included data.table (version 1.18.2.1; data.table development team), readxl (version 1.4.5; Posit Software, PBC, Boston, MA, USA), NMF (version 0.28; University of Cape Town, Cape Town, South Africa, and National University of Ireland Galway, Galway, Ireland), survival (version 3.8-6; Terry M. Therneau, Mayo Clinic, Rochester, MN, USA), survminer (version 0.5.2; Alboukadel Kassambara, Datanovia, Marvejols, France), pheatmap (version 1.0.13; Raivo Kolde, University of Tartu, Tartu, Estonia), ComplexHeatmap (version 2.22.0; German Cancer Research Center, Heidelberg, Germany), ggplot2 (version 4.0.2; Posit Software, PBC, Boston, MA, USA), dplyr (version 1.2.0; Posit Software, PBC, Boston, MA, USA), preprocessCore (Ben Bolstad), and quadprog (Berwin A. Turlach, University of Western Australia, Perth, Australia). Institutional locations refer to the supporting organizations or developers’ affiliations. Plots were generated using ggplot2, ComplexHeatmap, pheatmap, and survminer.

## 3. Results

### 3.1. The Immune Cell Protein Reference Matrix Preserves Lineage and Cell State Specificity

**Figure 1:**
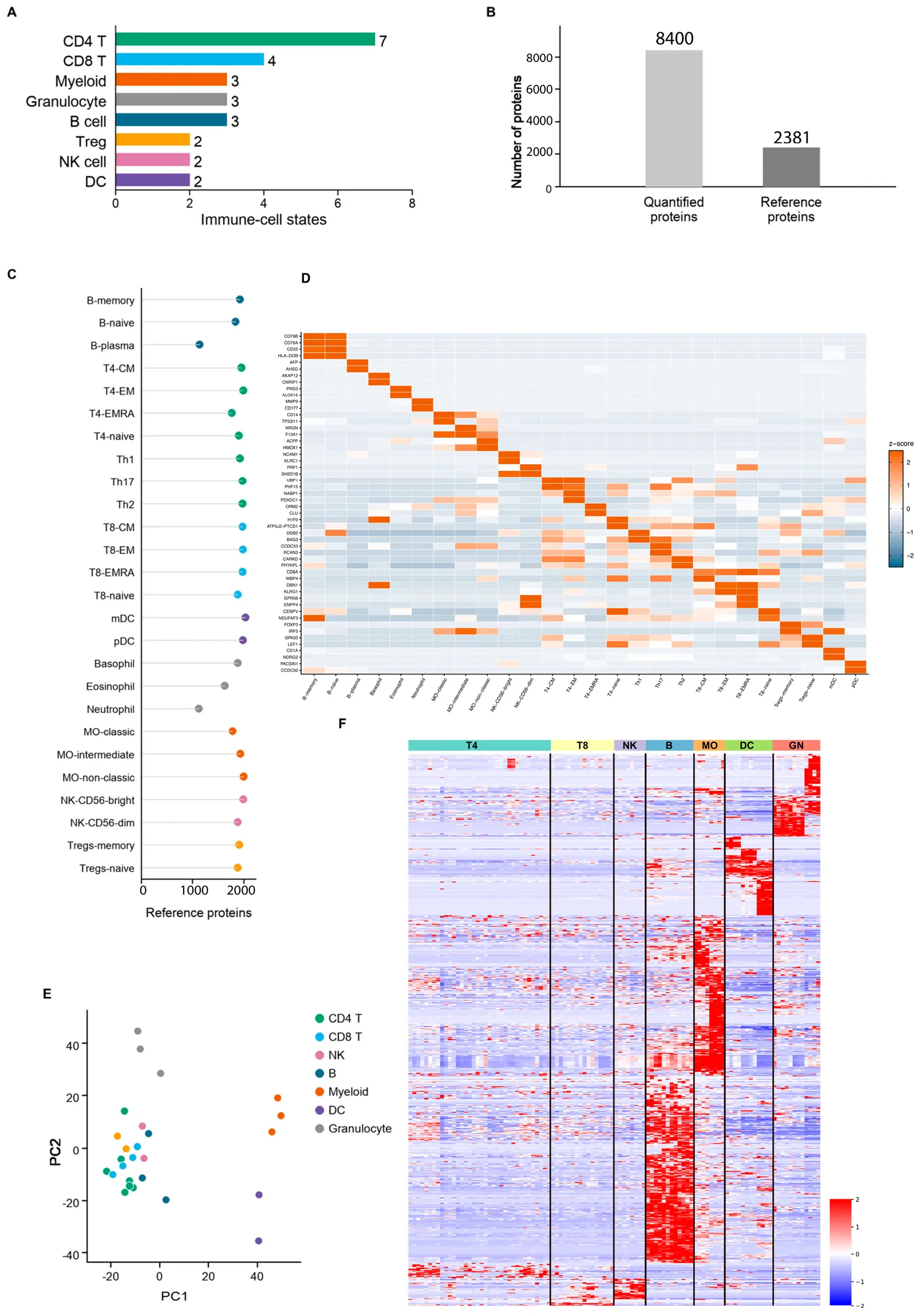
Construction and characterization of the immune-cell proteomic reference matrix. (**A**) Distribution of the 26 immune-cell states across the immune-cell categories represented in the reference dataset. The number at the end of each bar indicates the number of cell states assigned to each category. (**B**) Protein coverage of the immune-cell reference dataset. A total of 8400 proteins were quantified, of which 2381 were retained in the curated reference matrix used for downstream modeling. (**C**) Number of reference proteins detected for each of the 26 immune-cell states. Colors denote immune-cell categories. (**D**) Heatmap of representative cell-state-selective proteins across the 26 immune-cell states. Rows represent proteins and columns represent immune-cell states; warmer colors indicate relatively higher abundance and cooler colors indicate relatively lower abundance after protein-wise standardization. (**E**) Principal-component analysis of immune-cell reference proteomes. Each point represents an immune-cell state and is colored according to its immune-cell category. (**F**) Global heatmap of the 2381 reference proteins across the 26 immune-cell states. Rows represent proteins and columns represent immune-cell states; values were standardized across cell states for visualization and displayed on a scale from −2 to 2. B, B cell; DC, dendritic cell; MO, monocyte; NK, natural killer cell; CM, central memory; EM, effector memory; EMRA, effector memory re-expressing CD45RA.

To infer immune-related states from the bulk proteome of lung adenocarcinoma, we first constructed and systematically evaluated a human immune cell protein reference matrix. This reference matrix covers 7 major immune lineages and 26 immune cell states, including major immune cell populations such as CD4 T cells, CD8 T cells, B cells, NK cells, myeloid cells, granulocytes, and dendritic cells, providing a relatively comprehensive cell-type foundation for subsequent immune state inference (Figure 1A). In terms of protein coverage, 8400 quantifiable proteins were detected in the reference data; after screening, 2381 reference proteins were retained for subsequent modeling (Figure 1B). Further evaluation of protein coverage by cell state revealed that all immune cell states retained a sufficient number of reference proteins, indicating that the matrix does not rely on a small number of individual markers but rather characterizes immune cell states based on multi-protein abundance patterns (Figure 1C).

We evaluated whether this reference matrix could preserve immune cell lineage and state specificity. Heatmaps of marker protein abundance by cell state showed that different immune cell states exhibit distinguishable protein abundance patterns, with multiple marker proteins showing relatively high abundance in specific immune cell states (Figure 1D).

Proteins associated with certain B cells, T cells, myeloid cells, and dendritic cells form locally high-abundance protein clusters within their respective cellular states, suggesting that this reference matrix can capture protein characteristics at the cellular state level. Principal component analysis further revealed that immune cell samples exhibit a certain degree of lineage separation in the protein abundance space, with different immune lineages tending to have relatively independent distributions in the low-dimensional space (Figure 1E). The overall reference protein abundance heatmap also reveals structured abundance patterns, with clear protein abundance differences between different immune cell lineages and cell states (Figure 1F).

To confirm the technical stability of the reference matrix, we conducted supplementary quality control analyses. As reference samples were progressively incorporated, the cumulative number of detected proteins increased rapidly and gradually plateaued, indicating that the current reference data already cover the major detectable immune cell protein space (Figure S1A). The abundance levels of reference proteins span a wide dynamic range, suggesting that the matrix can capture both high- and low-abundance protein signals simultaneously (Figure S1B). Sampling stability analysis showed that the coefficient of variation gradually decreased as the proportion of included samples increased, indicating that the reference matrix exhibits good detection stability at the sample level (Figure S1C).

The immune cell protein reference matrix provides comprehensive coverage of cellular states, sufficient protein detection depth, and well-defined lineage specificity, and can serve as the foundation for inferring the immune status of bulk proteins in lung adenocarcinoma.

### 3.2. Establishing a Computational Framework for Lung Adenocarcinoma Immune Status Based on Proteomic Matrices

**Figure 2:**
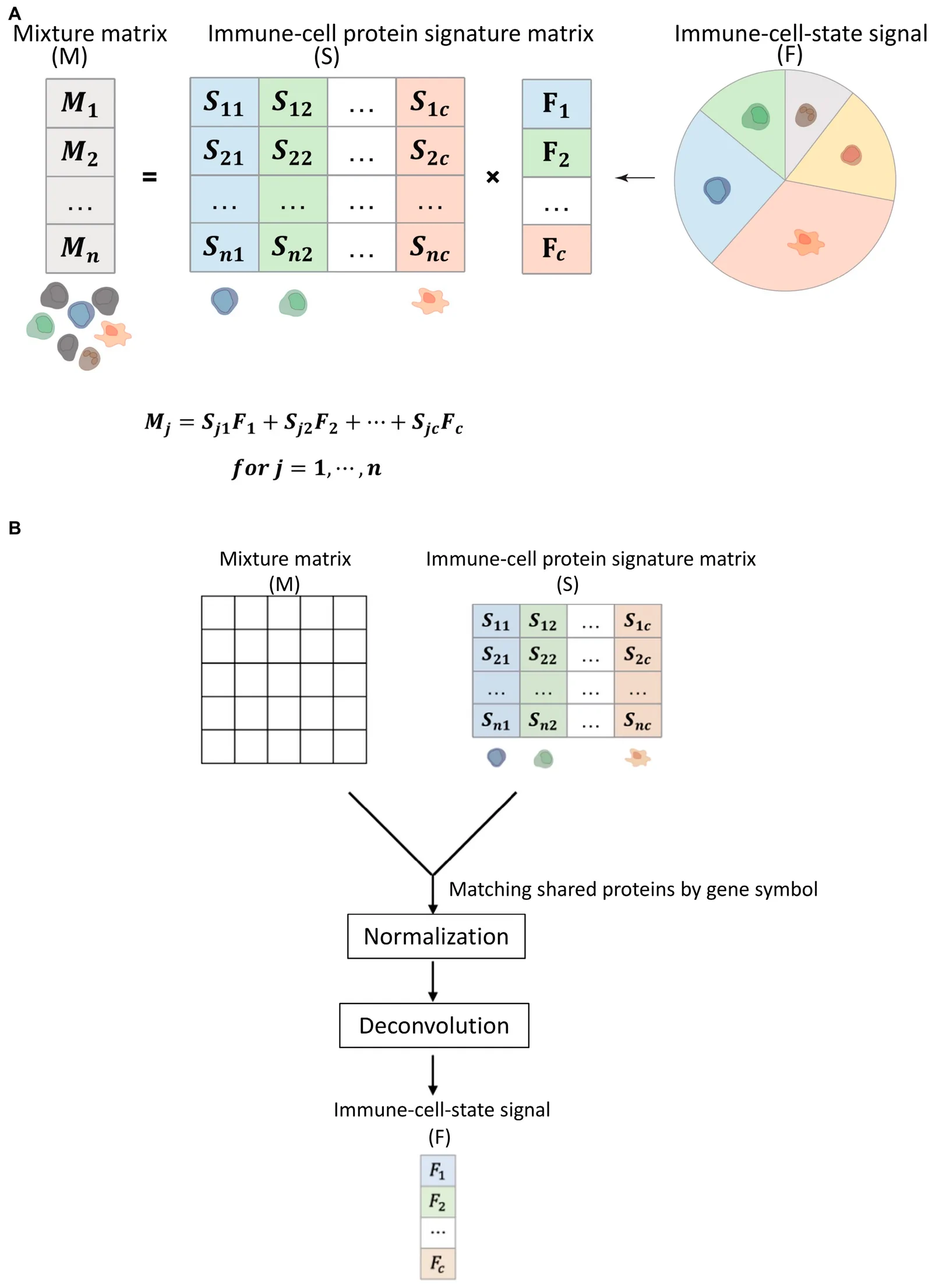
Computational framework for estimating protein-defined immune-cell-state signals from bulk LUAD proteomes. (**A**) Mathematical representation of the deconvolution model. For each bulk tumor sample, the tumor protein abundance vector (*M*) is modeled as the product of the immune-cell protein signature matrix (*S*) and the immune-cell-state signal vector (*F*), such that (*M_j_* $=\;{\mathrm{sum}}_{c\;=\;1}^{C}S_{\mathrm{jc}}F_{c}$, where (*j*) indexes proteins and (*c*) indexes immune-cell states. The coefficients in (*F*) quantify the relative contribution of reference immune-cell-state protein abundance patterns to the bulk tumor proteome and should not be interpreted as absolute histological cell fractions. (**B**) Analysis workflow. The LUAD bulk-tumor proteomic matrix and immune-cell protein signature matrix were harmonized at the gene-symbol level and restricted to their shared protein feature space. Non-negative constrained regression was then used to estimate the 26 immune-cell-state signals for each tumor sample. Non-negative coefficients were normalized within each sample so that their sum equaled one. The resulting 26-dimensional protein-defined immune-state profile was used for downstream sample comparison, NMF-based subtyping, and survival analyses. LUAD, lung adenocarcinoma; NMF, non-negative matrix factorization.

After confirming that the immune cell protein reference matrix possesses the ability to distinguish cell states, we established a computational framework for immune status based on bulk tumor proteomics. The core idea of this framework is to map the lung adenocarcinoma tumor protein abundance matrix and the immune cell reference protein matrix to a common protein feature space, and to use the reference matrix to estimate the strength of protein signals associated with different immune cell states in each tumor sample. We unified the tumor protein abundance matrix and the immune cell reference matrix at the gene symbol level and selected proteins common to both as inputs for subsequent modeling to ensure that tumor samples and the immune cell reference matrix could be compared within the same feature space (Figure 2A).

The bulk tumor mixture matrix was quantile-normalized across samples before intersection with the immune-cell reference matrix. The reference matrix was globally standardized across all proteins and immune-cell states, whereas each tumor sample was independently z-score standardized across the shared proteins. Non-negative constrained regression was then used to estimate the 26 immune-cell-state signals. The fitted coefficients were normalized within each sample so that their sum equaled one. These signals reflect the relative protein abundance patterns associated with different immune-cell states in the bulk tumor proteome and do not represent the absolute proportions of immune cells in the tissue. This framework represents each lung adenocarcinoma sample as a 26-dimensional protein-based immune-state profile, providing a quantitative basis for subsequent sample classification and prognostic analysis.

### 3.3. Significant Protein-Defined Immune Status Heterogeneity Exists in the Lung Adenocarcinoma Cohort

**Figure 3:**
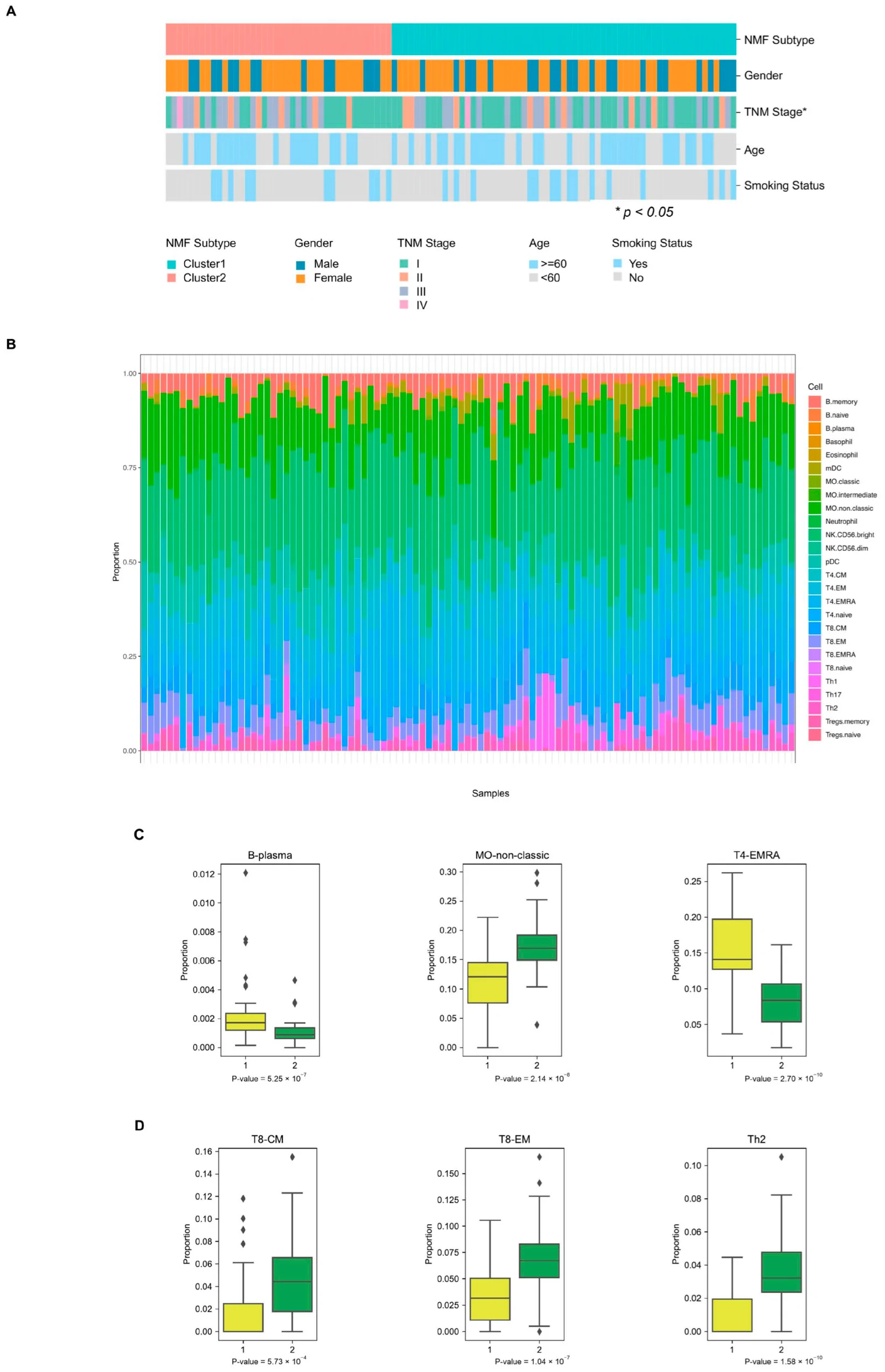
Proteomic immune-state heterogeneity and immune-subtype-associated differences in the LUAD discovery cohort. (**A**) Clinical annotation map for the 101 LUAD tumors, ordered by the two NMF-derived immune subtypes. Annotation tracks show subtype assignment, sex, TNM stage, age group, and smoking status. The asterisk denotes a nominal association with sub-type at *p* < 0.05. (**B**) Relative composition of the 26 protein-defined immune-cell-state signals across individual LUAD tumors. Each bar represents one tumor and colored segments represent within-sample normalized contributions of the indicated immune-cell states; segments sum to one for each sample. These values represent relative proteomic similarity to reference immune-cell-state patterns rather than absolute cell proportions. (**C**,**D**) Distributions of representative immune-cell-state signals in Subtype 1 and Subtype 2. (**C**) B-plasma, MO-non-classic, and T4-EMRA signals. (**D**) T8-CM, T8-EM, and Th2 signals. Between-subtype comparisons were performed using two-sided Mann–Whitney U tests. Box centers indicate medians, boxes indicate interquartile ranges, whiskers extend to 1.5 times the interquartile range, and points beyond whiskers indicate outliers.

We applied the aforementioned protein-based immune status computational framework to 101 lung adenocarcinoma tumor proteomic samples. This main cohort has matched clinical annotation data, which can be used to characterize the sample background and evaluate the relationship between immune status and clinical outcomes (Figure 3A). At the protein level, the lung adenocarcinoma tumor proteome matrix and the immune cell reference matrix exhibit sufficient overlap in protein coverage, supporting further inference of protein-based immune status.

Based on scores for 26 protein-defined immune cell states, we observed distinct differences in immune state composition among lung adenocarcinoma samples (Figure 3B). The contribution of scores for lymphocyte-related, myeloid-related, granulocyte-related, and dendritic cell-related states varied across samples, suggesting significant immunomicroenvironmental heterogeneity in lung adenocarcinoma tumor tissues at the proteomic level. Some samples exhibited higher T-cell, B-cell, or NK-cell-related signals, while others showed enhanced myeloid or granulocyte-related states.

To further elucidate this heterogeneity, we compared the distribution of representative immune cell states across the two protein-based immune subtypes. The results showed differences in multiple immune cell state scores between the two subtypes (Figure 3C,D). These differences were not confined to a single immune cell state but involved multiple immune lineage-related states, suggesting that the proteomic immune subtypes of lung adenocarcinoma are collectively shaped by multiple immune cell states. Supplementary analyses further demonstrated the protein coverage of the main cohort of lung adenocarcinoma and the distribution of clinical variables such as TNM staging, age, sex, and smoking status (Figure S2A–E). Additionally, the scores for all 26 immune cell states across the two subtypes are presented (Figure S2F–AC).

Together, these results demonstrate that the bulk proteome of lung adenocarcinoma can capture differences in immune microenvironment-related protein signals across samples and support further unsupervised classification based on protein-based immune states.

### 3.4. Proteomics-Supported Immune Cell Infiltration Identifies Lung Adenocarcinoma Subtypes Significantly Associated with DFS

**Figure 4:**
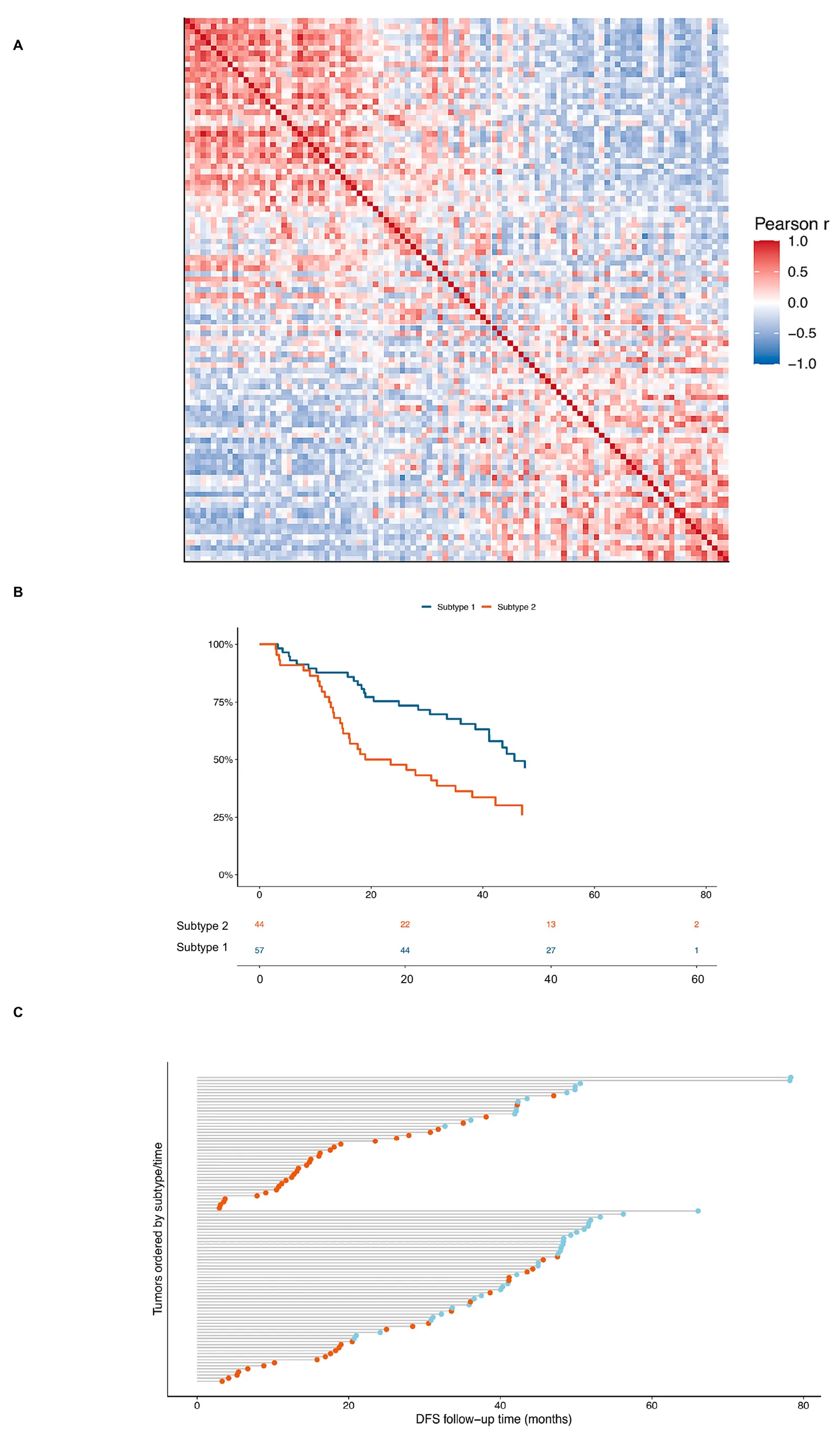
Protein-defined immune subtypes are associated with disease-free survival in the LUAD discovery cohort. (**A**) Pairwise sample-similarity matrix calculated using Pearson correlation coefficients across the 26 protein-defined immune-cell-state signals. Rows and columns represent the 101 LUAD tumors, ordered by NMF-derived subtype; red indicates positive correlation and blue indicates negative correlation. (**B**) Kaplan-Meier estimates of disease-free survival for Subtype 1 (n = 57) and Subtype 2 (n = 44). The y-axis indicates the estimated disease-free survival probability, and the x-axis indicates follow-up time in months. Differences were evaluated using a two-sided log-rank test (p = 0.0047); numbers at risk are shown below the plot. In univariable Cox proportional hazards analysis, Subtype 2 was associated with a higher risk of a DFS event than Subtype 1 (HR, 2.09; 95% CI, 1.24-3.54; p = 0.0057). (**C**) Patient-level DFS follow-up in the discovery cohort, stratified by immune subtype and ordered by follow-up time within subtype. Horizontal lines represent follow-up intervals; orange points indicate DFS events and blue points indicate censored observations. DFS, disease-free survival; HR, hazard ratio.

To determine whether the aforementioned immune status heterogeneity constitutes a stable subtyping structure, we calculated Pearson correlations among 101 lung adenocarcinoma samples based on 26 immune cell status scores. A sample-to-sample similarity heatmap revealed that lung adenocarcinoma samples form two major similarity clusters based on protein-defined immune status, suggesting a structured separation of these tumor samples at the level of immune protein status (Figure 4A). Following unsupervised clustering based on this immune status structure, the samples were classified into two protein-defined immune subtypes.

We then evaluated the relationship between the two immune protein subtypes and disease-free survival (DFS). Kaplan–Meier analysis revealed that the DFS curves for the two subtypes were distinctly separated, with patients in Subtype 2 exhibiting significantly worse DFS than those in Subtype 1 (log-rank *p* = 0.0047; Figure 4B). Individual follow-up plots further demonstrated that DFS events were more concentrated and occurred at a higher rate in Subtype 2, whereas Subtype 1 had a relatively higher proportion of samples with longer DFS or no events (Figure 4C). This indicates that the protein-defined immune status not only reflects the heterogeneity of the lung adenocarcinoma immune microenvironment but also identifies patient subgroups with different DFS outcomes.

Further risk estimates showed that Subtype 2 had a higher risk of DFS events than Subtype 1. Among 101 lung adenocarcinoma samples, a total of 57 DFS events were observed. Subtype 1 comprised 57 cases, of which 26 experienced DFS events; Subtype 2 comprised 44 cases, of which 31 experienced DFS events. Univariate Cox analysis showed a significantly increased risk of DFS events in Subtype 2 (HR = 2.09, 95% CI 1.24–3.54; *p* = 0.0057). This association persisted after adjusting for clinical variables (HR = 1.87, 95% CI 1.06–3.30; *p* = 0.030). After further adjusting for mutation-related variables, Subtype 2 remained associated with a higher risk of DFS (HR = 2.01, 95% CI 1.08–3.72; *p* = 0.027). These results support the use of protein-defined immune status for DFS risk stratification in lung adenocarcinoma.

### 3.5. The CPTAC Cohort Validates That Immune Cell Infiltration Derived from Proteomics Supports LUAD Subtyping

**Figure 5:**
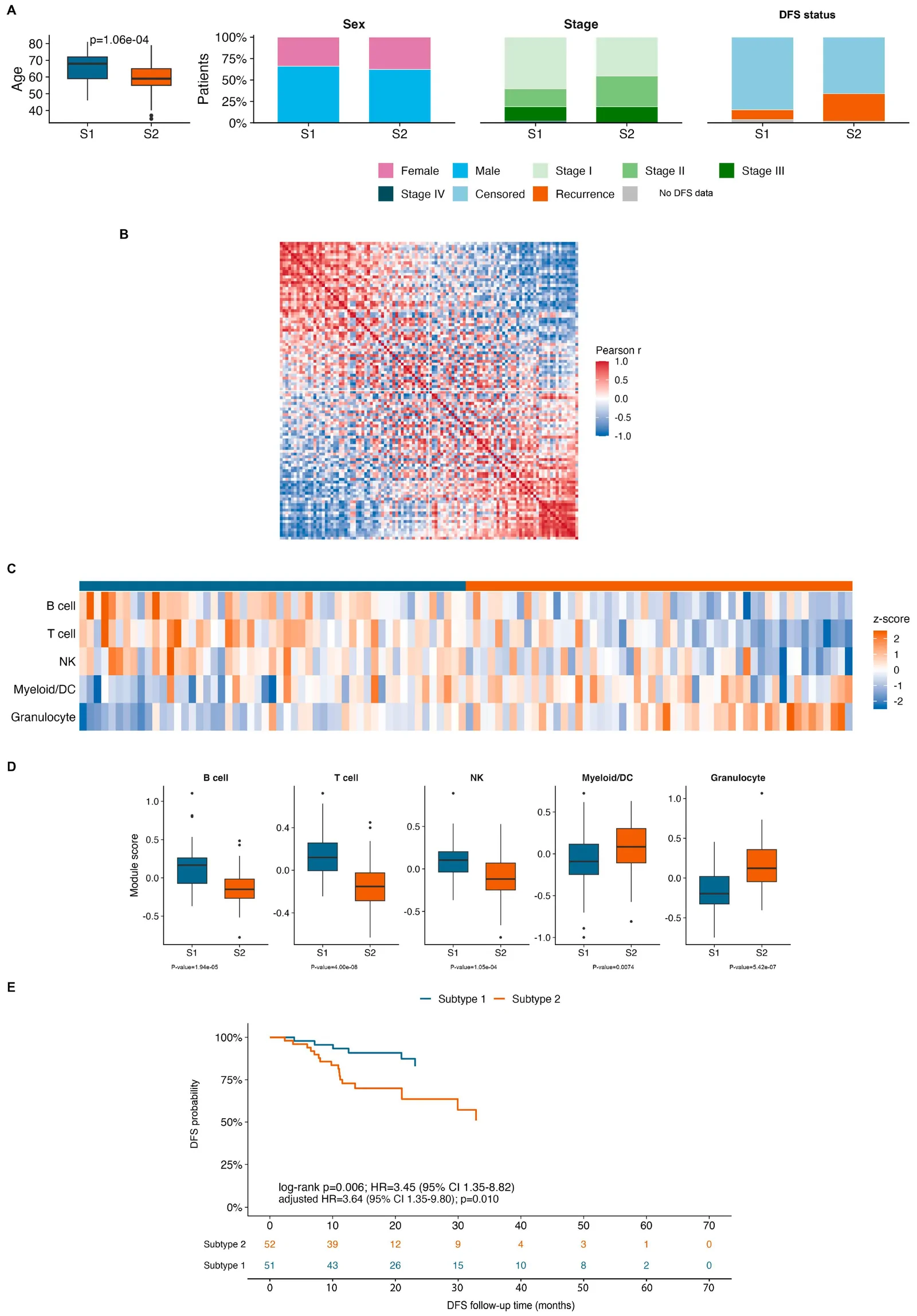
External validation of prognostic immune-protein subtypes in the CPTAC-LUAD cohort. (**A**) Clinical characteristics of the two marker-module-derived subtypes in the validation cohort. Age is shown as a box-and-whisker plot, whereas sex, pathological stage, and DFS status are shown as within-subtype proportions. The age difference was evaluated using a two-sided Mann–Whitney U test. (**B**) Pairwise Pearson correlation matrix among CPTAC-LUAD samples based on the five immune marker-protein module scores. Samples were ordered by rank-2 NMF subtype; red indicates positive correlation and blue indicates negative correlation. (**C**) Heatmap of z-score-normalized B-cell, T-cell, NK-cell, Myeloid/DC, and Granulocyte module scores across the validation cohort. Columns represent patients, rows represent immune marker-protein modules, and the top annotation indicates subtype assignment. (**D**) Distributions of the five immune marker-protein module scores by subtype. Subtype 1 showed relatively higher B-cell, T-cell, and NK-cell module scores, whereas Subtype 2 showed relatively higher Myeloid/DC and Granulocyte module scores. Between-subtype comparisons were performed using two-sided Mann–Whitney U tests with Benjamini-Hochberg correction. (**E**) Kaplan–Meier estimates of DFS for Subtype 1 (*n =* 51) and Subtype 2 (*n =* 52). Subtype 2 had significantly poorer DFS than Subtype 1 (log-rank *p =* 0.0061). In univariable Cox analysis, the HR for Subtype 2 versus Subtype 1 was 3.45 (95% CI, 1.35–8.82; *p =* 0.0098). After adjustment for age, sex, and pathological stage, the association remained significant (adjusted HR, 3.64; 95% CI, 1.35–9.80; *p =* 0.010). CPTAC, Clinical Proteomic Tumor Analysis Consortium; DFS, disease-free survival.

To assess whether the findings from the main cohort could be validated in an independent proteomics cohort, we further analyzed the CPTAC-LUAD validation cohort. This cohort features a processed tumor protein abundance matrix and clinical follow-up data suitable for DFS analysis, making it an appropriate external validation dataset (Figure 5A). We calculated the module score for each CPTAC-LUAD sample based on five classes of immune marker protein modules—B cell, T cell, NK, Myeloid/DC, and Granulocyte—and performed rank-2 NMF clustering based on these marker module scores. A Pearson correlation heatmap of sample-to-sample relationships shows that CPTAC-LUAD samples similarly form two relatively distinct immunoprotein similarity modules, indicating that immunoprotein states at the marker module level exhibit a distinguishable structure across independent cohorts (Figure 5B).

Further analysis of the immune module characteristics of the two validated subtypes revealed that Subtype 2 was primarily characterized by elevated levels of the Granulocyte and Myeloid/DC marker modules, whereas Subtype 1 was primarily characterized by elevated levels of the B cell, T cell, and NK marker modules (Figure 5C). Box-and-whisker plots further quantified these differences, showing systematic differences in the distribution of different marker modules between the two subtypes (Figure 5D). These results demonstrate that the prognostic-related subtyping in the external validation cohort exhibits distinct immunoprotein module characteristics, as follows: subtypes with poorer prognosis are more biased toward myeloid/granulocyte-related signaling, whereas subtypes with better prognosis are more biased toward lymphocyte- and NK-related signaling.

To further characterize the myeloid-associated signals, we examined macrophage-related proteins in the LUAD proteomic matrix. CD163 and MRC1, the official gene symbol for CD206, showed numerically higher median protein signals in Subtype 2 than in Subtype 1; however, neither difference was statistically significant. CD80 was not detected in the analyzed matrix, whereas CD86 was detected in only seven samples and did not show a significant subtype difference. These results do not support a definitive assignment of the increased myeloid-associated signal in Subtype 2 to a specific M1-like or M2-like macrophage state (Supplementary Table S1).

DFS survival analysis further supports the clinical significance of this marker module-based subtyping. In the CPTAC-LUAD validation cohort, Subtype 1 comprised 51 patients with 6 DFS events, while Subtype 2 comprised 52 patients with 17 DFS events. Kaplan–Meier analysis showed that DFS in Subtype 2 was significantly worse than in Subtype 1 (log-rank *p* = 0.0061; Figure 5E). Cox analysis revealed that Subtype 2 had a significantly higher risk of DFS events compared to Subtype 1 (HR = 3.45, 95% CI 1.35–8.82; Cox *p* = 0.0098). After adjusting for age, sex, and stage, the DFS risk for Subtype 2 remained significantly elevated (adjusted HR = 3.64, 95% CI 1.35–9.80; *p* = 0.010).

To further assess the robustness of the external validation strategy, we compared the relationship between various marker/module-based subtyping methods and DFS outcomes. The results showed that strategies such as marker module NMF, marker module hierarchical clustering, overall marker split, and NK marker split all exhibited the same risk trend, with marker module NMF selected as the primary strategy for external validation in the main text (Figure S3A).

Clinical annotations for individual samples and aggregated clinical variables in the validation cohort showed that Subtype 1 had better DFS, while Subtype 2 had more DFS events (Figure S4A,B). Individual DFS follow-up plots further demonstrated that DFS events in Subtype 2 were more concentrated, consistent with the separation from the DFS curve in the main text (Figure S4C).

These results demonstrate that protein-based immune subtypes defined by immune cell composition can identify patient subgroups with poor DFS outcomes in an independent lung adenocarcinoma proteomics cohort and support the external reproducibility of the findings from the main cohort.

## 4. Discussion

Based on a proteomic abundance matrix of immune cells, this study identified two immune subtypes of bulk lung adenocarcinoma with distinct immune protein characteristics and DFS outcomes. By integrating a reference matrix of immune cell proteins with a tumor protein abundance matrix for lung adenocarcinoma, we represented each tumor sample as a protein-immune profile consisting of 26 immune cell states and performed unsupervised clustering based on this profile. The results show that lung adenocarcinoma samples exhibit significant heterogeneity at the level of protein-defined immune states and can be grouped into two major immune subtypes. In the main cohort, Subtype 2 had significantly worse DFS than Subtype 1; in the independent CPTAC-LUAD proteomics cohort, a validation strategy based on immune cell marker protein modules similarly identified Subtype 2 as having a poorer DFS outcome. These results demonstrate that the lung adenocarcinoma proteome not only reflects intrinsic molecular alterations in tumor cells but also contains immune cell-related protein signals that can be used to characterize the state of the immune microenvironment and differences in patient prognosis.

The core finding of this study is that the lung adenocarcinoma proteome can define immunological subtypes with prognostic significance, and that this classification does not stem simply from a single immune cell marker but is jointly shaped by multiple protein programs related to immune cell states. In the main cohort, a sample similarity analysis based on 26 proteins defining immune cell state signals revealed that lung adenocarcinoma samples could be grouped into two abundance similarity modules, and these two modules corresponded to significantly different DFS outcomes. Furthermore, in the validation cohort, the marker protein module classification again identified two immune protein subtypes with different DFS outcomes, indicating that the classification structure observed in the main cohort exhibits a certain degree of external reproducibility. Since the validation cohort and the main cohort originated from different studies and different proteomics platforms, and exhibited differences in protein detection coverage and missing value patterns, these results support the conclusion that the immune protein subtypes are not a coincidental phenomenon arising from a single dataset or a single modeling process, but may reflect relatively stable immune microenvironment protein characteristics in LUAD.

From a biological perspective, Subtype 2 is primarily characterized by elevated levels of the granulocyte and myeloid/DC marker protein modules, accompanied by poorer disease-free survival (DFS) outcomes. Myeloid cells in the tumor microenvironment are highly heterogeneous, comprising both cell populations with antigen-presenting and immune-activating functions, as well as immunosuppressive myeloid cell populations that promote tumor progression, suppress T-cell responses, and attenuate the efficacy of immunotherapy [29]. Myeloid-derived suppressor cells, tumor-associated macrophages, and inflammatory myeloid cells can promote immune evasion by secreting immunosuppressive cytokines, modulating the metabolic environment, inhibiting T-cell activation, and reshaping the local inflammatory state within the tumor [20]. Furthermore, tumor-associated neutrophils and granulocyte-like myeloid cells have been associated with chronic inflammation, tumor progression, metastasis, and poor clinical outcomes in various solid tumors [30]. Therefore, the elevated Myeloid/Granulocyte-related protein module in high-risk Subtype 2 in this study may represent a pro-inflammatory, myeloid-enriched, or immunosuppressive tumor immune microenvironment, providing a plausible biological explanation for its poorer DFS outcomes.

However, CD163 and MRC1 showed only numerically higher signals in Subtype 2 without reaching statistical significance, while CD80 was not detected and CD86 had limited detection. Therefore, the increased myeloid-associated signal should not be interpreted as direct evidence of a specific M1-like or M2-like macrophage population. Future studies using larger cohorts, deeper proteomic coverage, and matched single-cell protein-level or CyTOF data may help distinguish more specific myeloid-cell states.

It is worth noting that the elevated Myeloid/DC module in Subtype 2 should not be simply interpreted as enhanced effective antigen presentation. Dendritic cells play a complex role in tumor immunity, as follows: on the one hand, classical dendritic cells can take up and present tumor antigens, promoting T-cell activation; on the other hand, under the influence of immunosuppressive factors, chronic inflammation, and abnormal metabolic stress in the tumor microenvironment, some dendritic cells may exhibit dysfunction or a state of tolerance, thereby impairing effective antigen presentation and antitumor T-cell responses [31]. The elevation of the Myeloid/DC module in the high-risk subtype within the validation cohort is more likely to reflect enhanced complex myeloid inflammation and immune regulation rather than simply representing an increase in protective antigen-presentation responses. This also suggests that future studies need to further integrate single-cell proteomics, spatial proteomics, or multiplex immunofluorescence techniques to clarify the true cellular origins and functional states of different myeloid/DC-related protein modules within the tissue space.

In contrast to Subtype 2, Subtype 1 is primarily characterized by elevated levels of B-cell, T-cell, and NK marker protein modules and is associated with relatively favorable disease-free survival (DFS) outcomes. T cells are central effector cells in antitumor immune responses; their activation, infiltration, and cytotoxic functions are closely linked to tumor immune surveillance, responses to immunotherapy, and the prognosis of various solid tumors. As important cytotoxic lymphocytes of the innate immune system, NK cells can recognize and eliminate tumor cells in a stressed state or with abnormal MHC-I expression, and regulate subsequent adaptive immune responses through cytokines and chemokines [32]. In recent years, B cells and tertiary lymphoid structures have been recognized as important components of the tumor immune microenvironment, often associated with more mature local antitumor immune responses, better clinical outcomes, and benefits from immunotherapy [4,33]. Elevated B-cell, T-cell, and NK-cell-related protein modules in Subtype 1 suggest that this subtype may represent an immune state of lung adenocarcinoma characterized by strong lymphocyte/NK-associated antitumor immune activity, which is consistent with its better DFS outcomes.

Another significant contribution of this study is that its inference of immune status is directly based on proteomic data. Transcriptomic approaches, including CIBERSORT, CIBERSORTx, xCell, MCP-counter, quanTIseq, EPIC, ESTIMATE, and TIMER, infer immune-cell abundance or activity from RNA-level marker signatures. These methods are highly valuable for analyzing large transcriptomic cohorts; however, RNA abundance does not always reflect protein abundance or protein activity. Protein-level measurements are influenced by translation efficiency, protein degradation, secretion, subcellular localization, and post-translational regulation, and may therefore provide complementary information about the functional state of the tumor immune microenvironment.

The present study did not include a matched transcriptomic matrix for the same 101 LUAD samples. Therefore, we did not perform a direct sample-level correlation analysis between the proteomic immune-cell-state signals and transcriptomic immune-infiltration estimates. The current findings should consequently be interpreted as a proteomic characterization rather than evidence that proteomic and transcriptomic immune classifications are either concordant or discordant in the same tumors.

Biologically, differences between RNA- and protein-based measurements may reflect post-transcriptional regulation, differences in protein stability, cellular secretion, and the functional activation state of immune cells. These differences may be informative rather than merely technical, because proteins are the direct effector molecules involved in antigen presentation, cytotoxicity, inflammatory signaling, chemokine activity, and immune regulation. From a diagnostic and prognostic perspective, proteomic and transcriptomic measurements should therefore be viewed as complementary rather than interchangeable. However, the clinical value of the proteomic immune-state signals requires further validation in matched multiomic cohorts and prospective studies.

CyTOF and bulk proteomics provide complementary views of the tumor immune microenvironment. CyTOF enables high-dimensional protein profiling at single-cell resolution and can distinguish immune-cell populations and phenotypic states using predefined antibody panels [34]. In contrast, the bulk proteomics framework used in this study does not resolve individual cells or provide absolute immune-cell proportions. Instead, it estimates relative protein-defined immune-cell-state signals from the combined tumor proteome. A potential advantage of bulk proteomics is its broader and relatively less marker-restricted protein coverage, which may capture coordinated immune-associated protein programs or cellular states that are not included in a predefined CyTOF panel. In addition, bulk proteomic datasets can be used for retrospective cohort-level analyses of existing clinical cohorts without requiring the collection of fresh single-cell samples. However, this broader coverage is accompanied by a loss of cellular resolution, and signals from multiple heterogeneous cell populations may be combined in the same bulk measurement. Therefore, the inferred immune-cell-state signals should not be interpreted as absolute cell proportions or as definitive functional states of individual immune-cell populations. The present approach should thus be considered complementary to CyTOF rather than a replacement for single-cell protein profiling. Future studies integrating matched CyTOF, single-cell proteomic, spatial proteomic, or multiplex immunofluorescence data may help determine how the inferred bulk-proteomic states correspond to specific cellular phenotypes.

The proteomic immune-cell-state signal obtained in this study is not equivalent to the absolute proportion of immune cells in the tissue; rather, it represents the relative protein signal intensity in the bulk proteome that matches the abundance patterns of reference proteins associated with the corresponding immune-cell states.

Validation in an independent CPTAC-LUAD cohort reinforces the credibility of this study’s findings. By integrating immune cell states into five major immune marker modules—B cell, T cell, NK, Myeloid/DC, and Granulocyte—the validation cohort could still be divided into two immune protein subtypes with significant differences in DFS. Furthermore, the high-risk subtype in the validation cohort similarly exhibited elevated levels of the Granulocyte and Myeloid/DC modules, while the low-risk subtype exhibited elevated levels of the B cell, T cell, and NK modules. These consistent immune module characteristics support the external reproducibility of the findings from the main cohort and suggest that this classification may capture an immune protein state that exists across cohorts, rather than a statistical grouping driven solely by a single algorithm or a single batch.

The number of immune-marker modules represents a balance between biological resolution and model stability. Using a larger number of modules could preserve finer immune-cell-state distinctions and potentially identify more specific cellular phenotypes. However, it would also increase model dimensionality and sensitivity to missing or low-abundance proteins, and could amplify platform-specific differences between cohorts. In contrast, using fewer modules could reduce noise and improve robustness but might oversimplify biologically distinct immune populations and obscure differences between related cell states. We therefore used five broad immune-lineage modules—B cell, T cell, NK, Myeloid/DC, and Granulocyte—as a compromise between resolution, detectability, and interpretability. This five-module representation provided a stable framework for external validation while preserving the major immune-lineage differences associated with prognosis.

Nevertheless, the five-module strategy cannot resolve all fine immune-cell states, and future studies using larger cohorts and matched single-cell protein or spatial proteomic data may help determine whether more granular module definitions provide additional prognostic information.

This study still has several limitations. First, the sample sizes of the main and validation cohorts are relatively limited; in particular, after subtype stratification and DFS analysis, the number of events within each subtype remains small. Therefore, further validation is needed using a larger-scale LUAD proteomics cohort with standardized follow-up information.

Although the original LUAD proteogenomic study reported clinical and molecular characteristics, including selected gene mutation information [23], matched tumor mutational burden (TMB) values and complete mutation-level data were not available for the 101 proteomic samples analyzed in the present study. Therefore, we could not reliably compare TMB between the two proteomic immune subtypes or evaluate its association with the inferred immune-cell-state signals. Future studies integrating matched genomic and proteomic data will be needed to clarify the relationships among TMB, neoantigen-related immunogenicity, and proteomic immune microenvironmental states.

Moreover, a matched transcriptomic dataset was not available for the 101 LUAD samples analyzed in this study. Therefore, the relationship between the proteomic immune-cell-state signals and transcriptomic immune-infiltration estimates could not be directly assessed in the same tumors. In addition, the current analysis was performed primarily at the canonical protein/gene-symbol level and did not systematically resolve proteoforms or peptide-specific post-translational modifications. Future matched multiomic studies will be required to determine how transcript-level, canonical protein-level, and proteoform-level measurements jointly contribute to immune-state classification and prognostic assessment.

Second, this study is a retrospective analysis of public data; although external validation was performed using an independent CPTAC-LUAD cohort, it cannot replace prospective clinical studies. Third, differences in detection depth, missing value rates, and quantification scales exist across different proteomics platforms; consequently, the validation cohort did not fully replicate the 26 immune cell infiltration ratios observed in the main cohort. Fourth, this study is primarily based on computational inferences and lacks experimental validation via immunohistochemistry, multiplex immunofluorescence, flow cytometry, or spatial proteomics; thus, the specific immune cell composition, spatial distribution, and functional states within each subtype cannot be directly confirmed. Fifth, the protein-defined immune cell state signals reflect bulk protein signals similar to those of reference immune cell protein patterns; therefore, they may be influenced by tumor purity, stromal components, protein missing values, and tissue processing methods. These considerations are also relevant to LUAD studies focused on stromal-cell infiltration and cancer-associated fibroblast-related prognostic features [3]. Future studies should integrate spatialomics, single-cell omics, and experimental pathological validation to further elucidate the cellular composition and functional mechanisms underlying these immunoprotein subtypes.

This study demonstrates that the bulk proteome of lung adenocarcinoma contains immune-cell-associated protein signals that reflect immune microenvironment heterogeneity and differences in DFS outcomes. The immune status inferred from the proteome can classify LUAD patients into two immunological subtypes with different prognoses, as follows: Subtype 2, which has a poorer prognosis, is characterized by elevated granulocyte- and myeloid/DC-related protein modules, while Subtype 1, which has a better prognosis, is characterized by elevated B-cell-, T-cell-, and NK-related protein modules. This classification was validated in an independent CPTAC-LUAD cohort, suggesting that immunophenotyping at the proteomic level may serve as a valuable complement to the analysis of the LUAD immune microenvironment and DFS risk stratification.

## Data Availability

The publicly available immune-cell proteomic dataset analyzed in this study was obtained from the ProteomeXchange Consortium via the PRIDE repository under accession number PXD004352. The LUAD discovery-cohort proteomic and clinical data were obtained from ProteomeXchange under accession number PXD020252. The CPTAC-LUAD validation-cohort proteomic data are available through the NCI Proteomic Data Commons under study accession PDC000153, and the associated genomic and clinical annotation data are available through the NCI Genomic Data Commons and dbGaP under study accession phs001287. Supplementary Data 05, containing the CPTAC-LUAD validation data used in this study, is publicly available at GitHub: https://github.com/RuoxianZhang/Immune-Cell-Infiltration. (accessed on 22 September 2026). The remaining processed data and analysis code generated or used in this study, including the immune-cell protein signature matrix, immune-cell-state scores, marker-protein lists, module scores, subtype assignments, survival-analysis input data, and source data underlying the figures, are available from the corresponding author upon reasonable request (Jun Qin, J.Q.; email: jqin1965@126.com), subject to institutional regulations, data-use restrictions, and intellectual-property considerations. The original public datasets can be obtained from the sources cited above and in the manuscript.

## Supplementary Materials

The following supporting information can be downloaded at: https://www.mdpi.com/article/10.3390/proteomes14040052/s1, Figure S1: Quality-control assessment of the immune-cell proteomic reference dataset. (A) Cumulative protein detection curve across immune-cell reference samples. The x-axis shows the sequentially added reference samples, and the y-axis shows the cumulative number of detected proteins. Colors denote immune-cell lineages. (B) Rank-abundance curves of protein intensity in the immune-cell reference dataset. The x-axis shows protein rank ordered by abundance, and the y-axis shows log10-transformed iFOT intensity. Curves are shown for the full reference dataset and major immune-cell lineages. (C) Sampling stability analysis of the immune-cell reference proteome. The x-axis shows the proportion of reference samples included in repeated subsampling, and the y-axis shows the coefficient of variation. Boxes indicate interquartile ranges and whiskers indicate dispersion across repeated sampling. Figure S2: Additional characterization of the LUAD discovery cohort and immune-cell-state signals. (A) Number of proteins detected before and after filtering in the LUAD discovery cohort. (B) Distribution of TNM stages among the 101 LUAD tumors. (C) Age distribution by sex. (D) Sex distribution of the LUAD cohort. (E) Smoking-status distribution of the LUAD cohort. (F–AC) Distributions of all 26 protein-defined immune-cell-state signals between Subtype 1 and Subtype 2. The x-axis shows immune subtype and the y-axis shows the normalized protein-defined immune-cell-state signal. Between-subtype comparisons were performed using two-sided Mann–Whitney U tests. Box centers indicate medians, boxes indicate interquartile ranges, whiskers extend to 1.5 times the interquartile range, and points beyond whiskers indicate outliers. Figure S3: Sensitivity analysis of external validation strategies in the CPTAC-LUAD cohort. (A) Forest plot comparing different marker-based external validation strategies. The x-axis shows the HR for DFS events in Subtype 2 versus Subtype 1, and the y-axis lists the subtype-definition strategies, including marker module NMF, marker module hierarchical clustering, overall marker split, and NK marker split. Points represent HR estimates and horizontal lines represent 95% confidence intervals. The dashed vertical line indicates HR = 1. HR values greater than 1 indicate higher DFS-event risk in Subtype 2. Figure S4: Clinical annotation and patient-level DFS follow-up in the CPTAC-LUAD validation cohort. (A) Sample-level clinical annotation map for the CPTAC-LUAD validation cohort, ordered by marker-module-derived subtype. Annotation tracks show subtype assignment, sex, pathological stage, age group, and DFS status. Each column represents one tumor sample. (B) Summary of clinical characteristics by subtype. Age is shown as a box-and-whisker plot, whereas sex, pathological stage, and DFS status are shown as stacked proportion plots. (C) Patient-level DFS follow-up in the validation cohort, stratified by subtype and ordered by follow-up time within subtype. Horizontal lines represent follow-up intervals; orange points indicate DFS events and blue points indicate censored observations. DFS, disease-free survival. Table S1: Macrophage-associated protein markers in the LUAD discovery cohort. The table summarizes the detection status, detection rate, subtype-specific median protein signals, and two-sided Mann–Whitney U test results for CD80, CD86, CD163, and MRC1/CD206. CD206 was evaluated using its official gene symbol MRC1. The reported values represent bulk proteomic signals and should not be interpreted as absolute macrophage abundance or cell proportions. Supplementary Data 05 is publicly available at GitHub: https://github.com/RuoxianZhang/Immune-Cell-Infiltration. (accessed on 22 September 2026).

## Abbreviations

The following abbreviations are used in this manuscript:

LUAD	Lung adenocarcinoma
NSCLC	Non-small cell lung cancer
DFS	Disease-free survival
CPTAC	Clinical Proteomic Tumor Analysis Consortium
NK	Natural killer
DC	Dendritic cell
NMF	Non-negative matrix factorization
HR	Hazard ratio
CI	Confidence interval
FDR	False discovery rate
TNM	Tumor–node–metastasis
RNA	Ribonucleic acid
mRNA	Messenger RNA
RNA-seq	RNA sequencing
iBAQ	Intensity-based absolute quantification
iFOT	Intensity-based fraction of total
PRIDE	Proteomics Identifications Database
NCBI	National Center for Biotechnology Information
MHC-I	Major histocompatibility complex class I
EGFR	Epidermal growth factor receptor
TP53	Tumor protein p53
KRAS	Kirsten rat sarcoma viral oncogene homolog
CD4	Cluster of differentiation 4
CD8	Cluster of differentiation 8
MCP-counter	Microenvironment Cell Populations-counter
EPIC	Estimating the Proportions of Immune and Cancer cells
ESTIMATE	Estimation of STromal and Immune cells in MAlignant Tumours using Expression data
