# Supplementary material for: Proteomic Profiling of Immune Cell Infiltration Reveals Prognostic Immune Subtypes in Lung Adenocarcinoma
Source: Proteomes. 2026 Oct 2;14(4):52. doi: 10.3390/proteomes14040052 (PMC13692338; doi:10.3390/proteomes14040052)
Supplement: Supplementary file 1 [file proteomes-14-00052-s001.zip › Supplementary/proteomes-4499906-supplementary.pdf]

Supplementary Figures

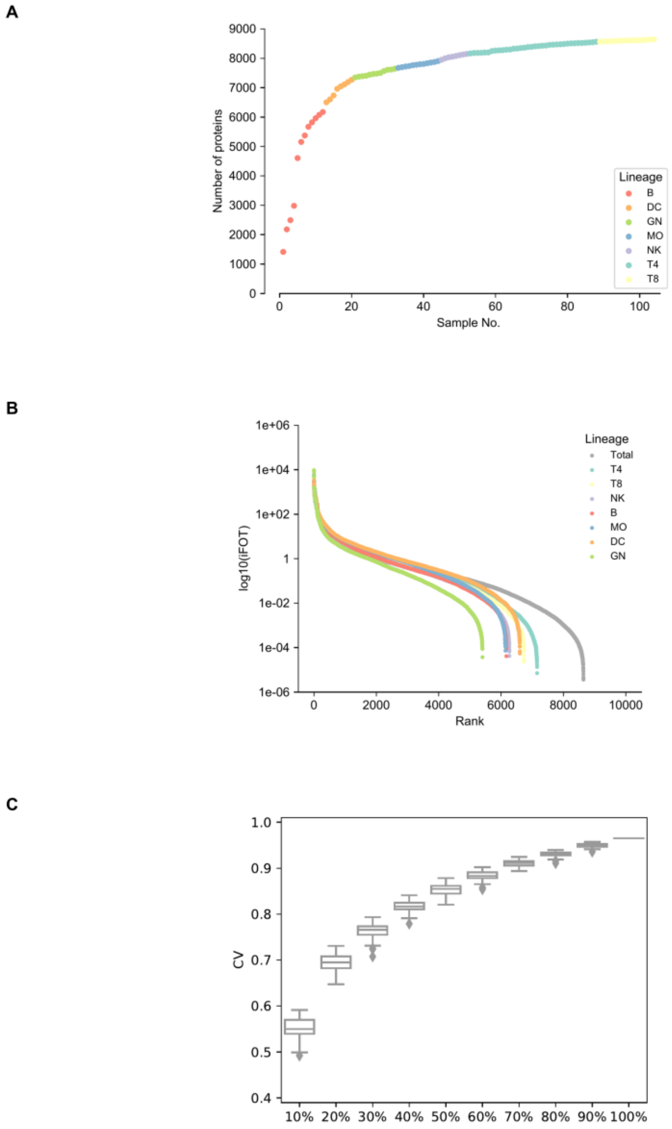

Figure S1. Quality-control assessment of the immune-cell proteomic reference dataset. (A) Cumulative protein detection curve across immune-cell reference samples. The x-axis shows the sequentially added reference samples, and the y-axis shows the cumulative number of detected proteins. Colors denote immune-cell lineages. (B) Rank-abundance curves of protein intensity in the immune-cell reference dataset. The x-axis shows protein rank ordered by abundance, and the y-axis shows log10-transformed iFOT intensity. Curves are shown for the full reference dataset and major immune-cell lineages. (C) Sampling stability analysis of the immune-cell reference proteome. The x-axis shows the proportion of reference samples included in repeated subsampling, and the y-axis shows the coefficient of variation. Boxes indicate interquartile ranges and whiskers indicate dispersion across repeated sampling.

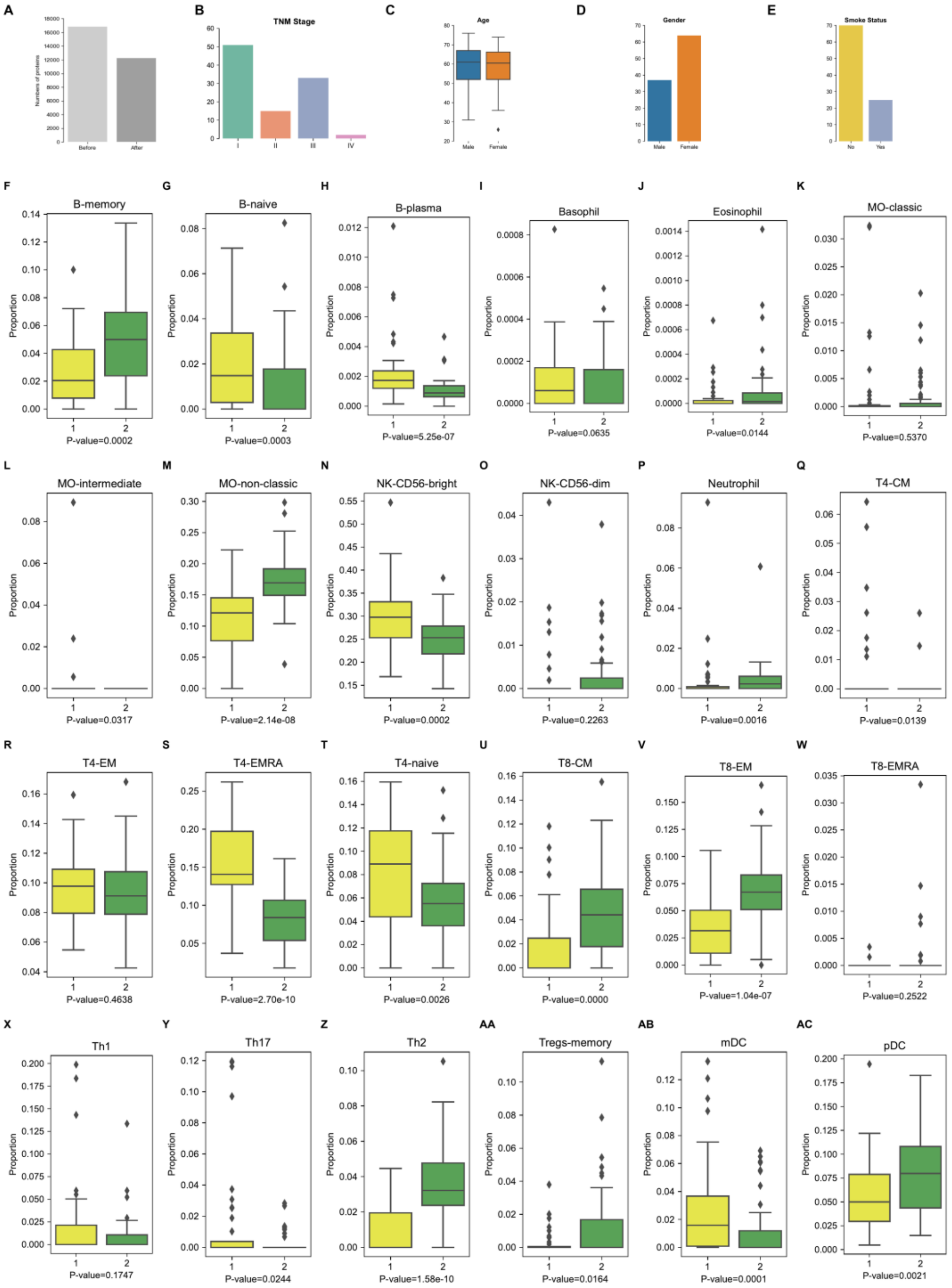

Figure S2. Additional characterization of the LUAD discovery cohort and immune-cell-state signals. (A) Number of proteins detected before and after filtering in the LUAD discovery cohort. (B) Distribution of TNM stages among the 101 LUAD tumors. (C) Age distribution by sex. (D) Sex distribution of the LUAD cohort. (E) Smoking-status distribution of the LUAD cohort. (F-AC) Distributions of all 26 protein-defined immune-cell-state signals between Subtype 1 and Subtype 2. The x-axis shows immune subtype and the y-axis shows the normalized protein-defined immune-cell-state signal. Between-subtype comparisons were performed using two-sided Mann-Whitney U tests. Box centers indicate medians, boxes indicate interquartile ranges, whiskers extend to 1.5 times the interquartile range, and points beyond whiskers indicate outliers.

A

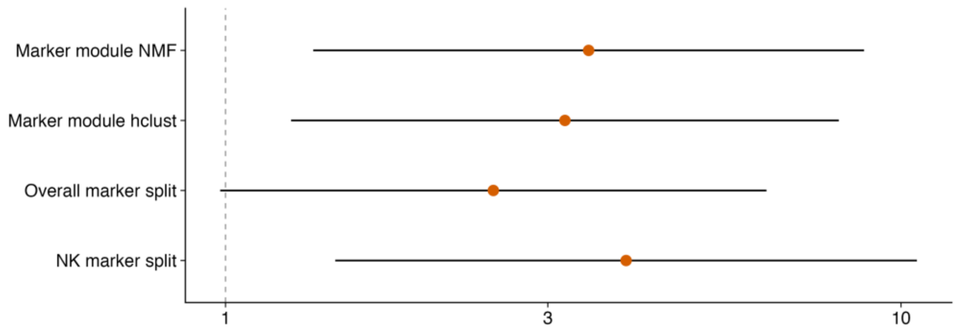

Figure S3. Sensitivity analysis of external validation strategies in the CPTAC-LUAD cohort. (A) Forest plot comparing different marker-based external validation strategies. The x-axis shows the HR for DFS events in Subtype 2 versus Subtype 1, and the y-axis lists the subtype-definition strategies, including marker module NMF, marker module hierarchical clustering, overall marker split, and NK marker split. Points represent HR estimates and horizontal lines represent 95% confidence intervals. The dashed vertical line indicates HR = 1. HR values greater than 1 indicate higher DFS-event risk in Subtype 2.

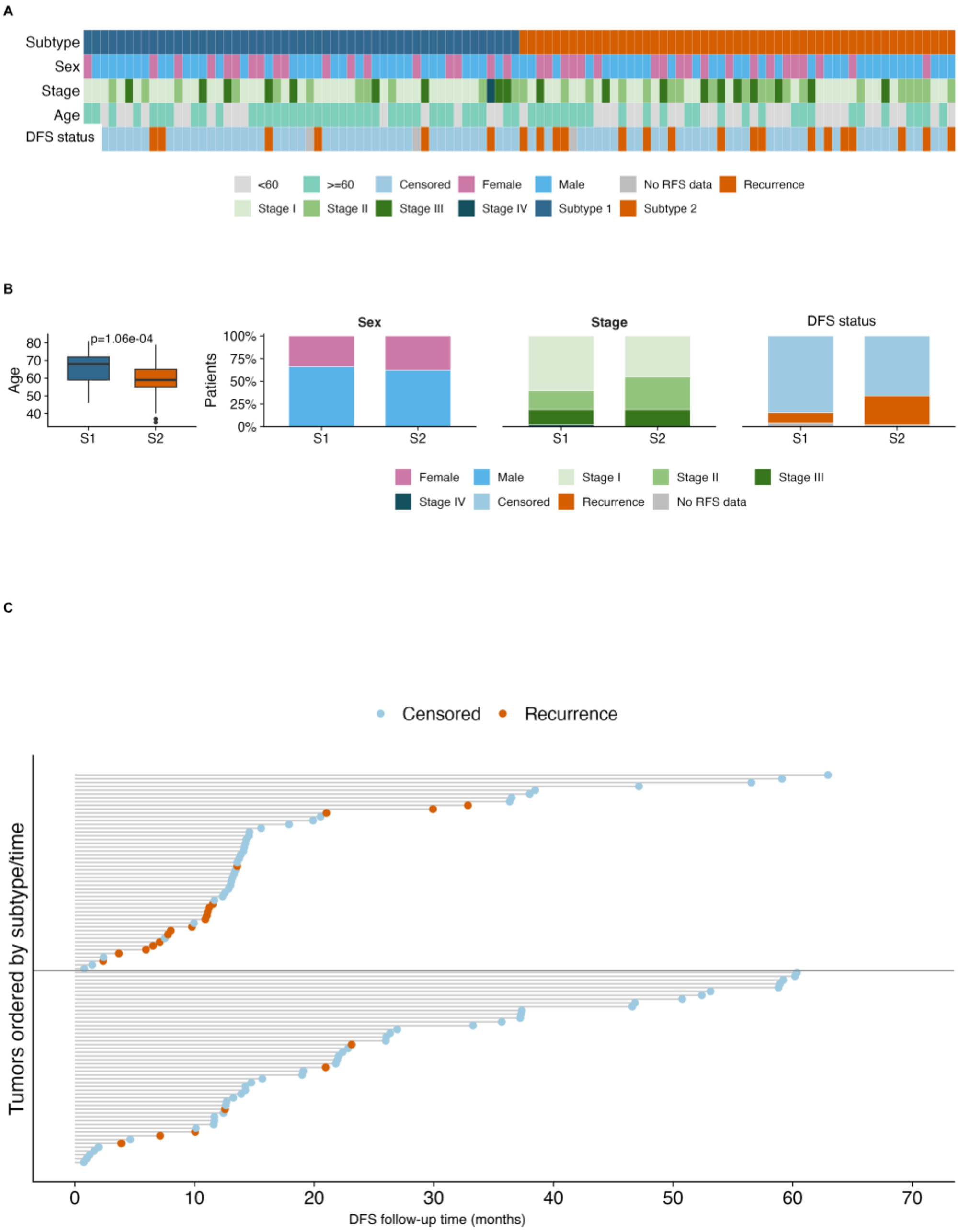

Figure S4. Clinical annotation and patient-level DFS follow-up in the CPTAC-LUAD validation cohort. (A) Sample-level clinical annotation map for the CPTAC-LUAD validation cohort, ordered by marker-module-derived subtype. Annotation tracks show subtype assignment, sex, pathological stage, age group, and DFS status. Each column represents one tumor sample. (B) Summary of clinical characteristics by subtype. Age is shown as a box-and-whisker plot, whereas sex, pathological stage, and DFS status are shown as stacked proportion plots. (C) Patient-level DFS follow-up in the validation cohort, stratified by subtype and ordered by follow-up time within subtype. Horizontal lines represent follow-up intervals; orange points indicate DFS events and blue points indicate censored observations. DFS, disease-free survival.
